# Supplementary material for: PI4P-Containing Vesicles from Golgi Contribute to Mitochondrial Division by Coordinating with Polymerized Actin
Source: Int J Mol Sci. 2023 Apr 1;24(7):6593. doi: 10.3390/ijms24076593 (PMC10095118; doi:10.3390/ijms24076593)
Supplement: Supplementary file 1 [file ijms-24-06593-s001.zip › ijms-2288595-supplementary.pdf]

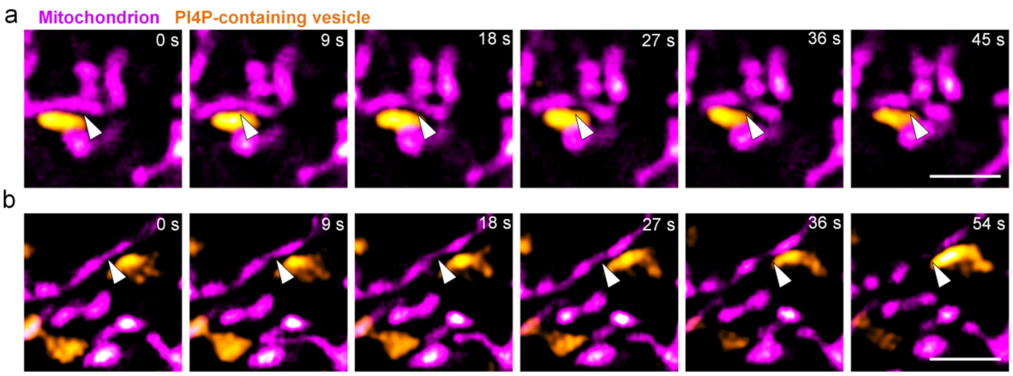

Supplementary Figure S1. PI4P-containing vesicles participate in mitochondrial division. a,b, PI4P-containing vesicles participate in mitochondrial division. White arrows indicate the site of mitochondrial division. Scale bar: 2 μm.

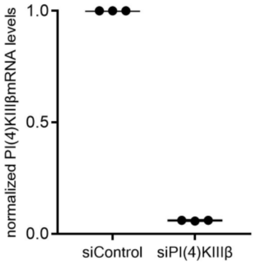

Supplementary Figure S2 RT-qPCR of siPI4KШβ. Normalized PI4KШβ mRNA levels in cells transfected with a siRNA specifically targeting PI4KШβ or a control siRNA, as measured by RT-qPCR. The graphs show the mean ± SEM, on the basis of cells from three independent experiments.

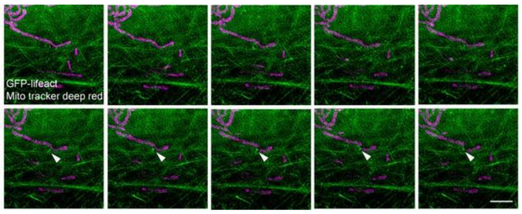

Supplementary Figure S3 Actin participates in mitochondrial division. SIM images showing actin (tagged with EGFP-Lifeact) and mitochondria (tagged with MitoTracker Deep Red) in live U-2 OS cells, aggregated actin clearly participated in mitochondrial division. White arrows indicate the site of mitochondrial division. Scale bar: 2 μm.
